# The 3-Minute Burpee Test: A Minimalistic Alternative to the Conventional Estimated Oxygen Uptake Test

**DOI:** 10.7759/cureus.35841

**Published:** 2023-03-06

**Authors:** Yohei Yamashita

**Affiliations:** 1 Sports Medicine, St. Andrew's University, Izumi, JPN

**Keywords:** yo-yo irt, field test, 3mbt, muscle strength, maximal oxygen uptake

## Abstract

Background

Maximal oxygen uptake and muscle strength are fundamental components of physical fitness. Improving these capacities is highly beneficial to health. The validity of maximal oxygen uptake and muscle strength has been widely emphasized in clinical, sports, and research-related settings. However, many of the previous tests required special equipment and space.

Aim

This study examined the effectiveness of field tests that do not require special equipment or space.

Materials and methods

The relationship between the 3-minute burpee test (3MBT) and estimated maximal oxygen uptake (Yo-Yo intermittent recovery test (Yo-Yo IRT)) using whole-body muscle groups was examined. The subjects were young men (n=127) with a history of exercising at least once a week.

Results

A strong relationship between 3MBT and Yo-YoIRT was shown (p<0.001).

Conclusions

The 3MBT is a field test that can be performed anytime and anywhere there is space for plank and standing postures. Because it is very brief, efficient, and uses muscle groups throughout the body, it is effective and potentially quite useful as a new field test.

## Introduction

Maximal oxygen uptake (V̇O2max) and strength abilities are the basic components of physical fitness that contribute to significant health benefits.

There is ample scientific evidence to suggest that regular aerobic and resistance training is a highly effective strategy for managing and improving physical fitness, preventing fatigue, and motivating individuals to pursue their fitness goals [1].

Maximal oxygen uptake represents the physiological upper limit of oxygen utilization to generate energy during intense exercise that takes place until voluntary exhaustion [2-3]. Maximal oxygen uptake is widely recognized as the gold standard for cardiorespiratory fitness and is usually measured using the cardiopulmonary exercise test (CPET) in clinical, applied physiology, sports and exercise science, and guidelines are established by the American Thoracic Society (ATS) [2,4-7]. It is also commonly used to diagnose cardiovascular disease​​ ​and predict all-cause mortality In addition, exercise prescriptions can be developed and the effectiveness of exercise programs can be evaluated [8-17].

Therefore, the validity of maximal oxygen uptake values obtained from cardiopulmonary exercise testing is widely considered important in clinical, sports, and research-related settings. However, cardiopulmonary exercise testing is unsuitable because it requires not only equipment such as gas analyzers, but also physicians, nurses, and skilled experts, as well as time to study a large number of subjects.

Consequently, field tests, such as Cooper runs and shuttle runs, have been developed to evaluate a large number of subjects immediately in the field [18]. While most field tests are assessed by continuous exercise, many ball games and other activities are intermittent and are related to the athlete's ability to perform repetitions of intense exercise. For example, the quality of soccer has been found to be related to the amount of high-intensity running during the game [19-21]. Therefore, in a ball game, such as soccer, it is desirable to assess the ability to repeatedly perform intense exercises and have the ability to recover from intense exercise. It is on this basis that the Yo-Yo Intermittent Recovery Test (Yo-Yo IRT) was developed [22].

Furthermore, when maximal oxygen uptake is compared between a bicycle ergometer, which primarily uses lower extremity muscle strength, and a rowing ergometer, which uses not only lower extremity muscle strength but also the large muscle groups of the upper extremities, the bicycle ergometer is reported to show slightly higher values, but the difference is very small [23]. Therefore, it is possible that an exercise loading method using large whole-body muscle groups may yield very similar results to an exercise loading method using primarily the muscles of the lower extremity system.

Therefore, the purpose of this study is to examine the validity of an exercise and maximal oxygen uptake measurement method using whole-body muscle groups as an alternative to the typical field test using lower extremity muscle strength; that is, to examine the relationship between exercise (burpee) and maximal oxygen uptake using whole-body muscle groups.

Burpees are an exercise that utilizes muscle groups throughout the body. The burpee consists of a series of physical movements that begin and end with a standing squat. The burpee has become very popular among athletes, and various forms of testing have been developed, but its relationship to maximal oxygen uptake is not yet clear.

Previous reports have shown that the 3-minute burpee test (3MBT) is effective in accurately assessing muscular endurance in young women and that the number of burpee completions has a significant negative correlation with body weight and body mass index (BMI) [24].

Once the relationship between the burpee test and maximal oxygen uptake is clarified, it will be very beneficial in many settings because it can be measured simultaneously in a large number of people without requiring specific equipment or a large space.

## Materials and methods

Yo-Yo intermittent recovery test (Yo-Yo IRT)

**Figure 1:**
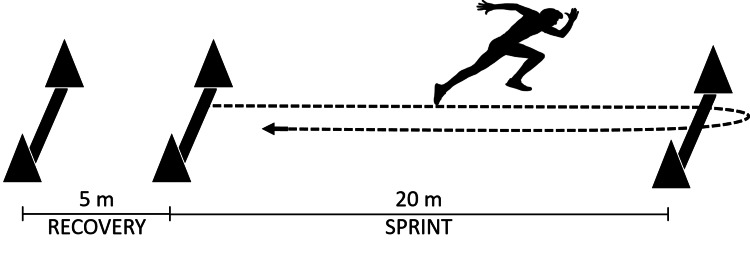
Yo-Yo intermittent recovery test (Yo-Yo IRT)

Yo-Yo IRT (Yo-Yo IRT L2, BangsboSport, Denmark), which was completed according to previously published protocols and involved players completing 2 × 20 m shuttle runs in time to an audio signal. Players started with both feet on or behind the first 20 m line and ran toward the second 20 m line aiming to reach this line in time with the audio signal. Players then turned and ran back to the starting line, again in time with the audio signal. At the end of each 2 × 20 m shuttle, there was a 10s period where players walked or jogged around a cone (placed 5 m past the finishing line) back to the starting line again for the next shuttle (Figure 1). The test was concluded if, after one warning, the player failed to complete a shuttle in time or the player removed themselves voluntarily. The Yo-Yo IRT L2 finishing shuttle number was converted to a distance and then used in the analysis.

3-minute burpee test (3MBT)

Burpees, invented by American biologist Royal Huddleston Burpee in the 1930s, are not only used in many sports and as military training but are also so popular that people around the world participate in online challenges held on the Internet [2-27]. Burpees are a multi-joint exercise that activates the major muscle groups of the body, including the pectoralis major, deltoids, quads, hamstrings, and trunk, and can be performed anywhere without a specific gym or equipment, in addition to being highly taxing on metabolic mechanisms [28-31].

**Figure 2:**
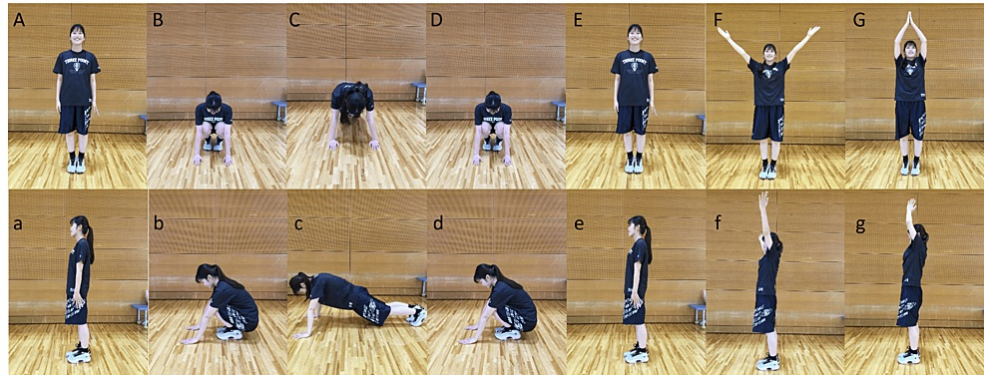
Burpee procedure Upper case letters represent frontal views and lower case letters represent lateral views. A through G was considered one cycle of burpees.

Burpees can be divided into four stages: squats, planks, push-ups, and jump-ups, but push-ups and jump-ups were excluded from this study. In addition, the burpee procedure consisted of one cycle of squat, plank, squat, and raising both hands above the head from a standing position (Figure 2).

**Figure 3:**
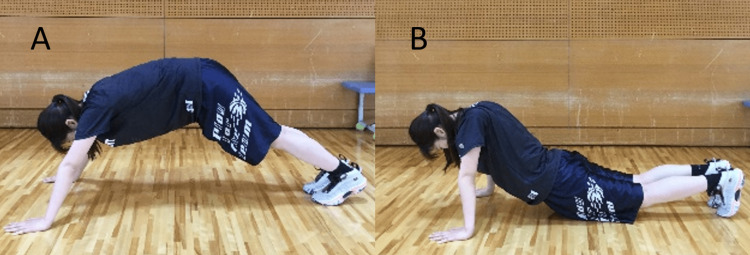
Examples of burpee failures A is an example where the hips are not lowered and the correct plank posture is not achieved. B is an example where the hips are too low and the correct plank posture cannot be achieved. If this occurs during the plank posture, it will not be counted.

The 3MBT was performed with particular attention to correct posture, rather than aiming for speed of repetition. If a subject failed to perform any one of the series of movements correctly, it was considered a failure and was not counted. In particular, a plank posture with high or low hips (Figure 3) and not clapping the hands over the head was also considered a failure.

In addition, subjects practiced the 3MBT five times a week, similar to the method used in previous studies, in order to increase the reliability of the 3MBT [30].

Prior to administering the test, subjects performed a 10-minute warm-up consisting of jogging, jumping jacks, mountain climbers, and stretching. These exercises were performed to increase muscle temperature in the pectoral, deltoid, quadriceps, hamstrings, and trunk muscles used in burpees. After five minutes of rest from the end of the warm-up, the 3MBT was begun.

A digital timer was used to measure the three minutes, and the number of cycles completed before the start and end buzzers sounded was used as the number of cycles performed.

Statistical analysis

The subjects were young men (n=127) who exercised once to three times a week for 90 minutes or less per session. All measures are expressed in mean (±SD). The significance level for all statistical tests was set at p < 0.05.

The relationship between 3MBT and Yo-Yo IRT was correlated using Pearson's product rate correlation coefficient (r). Interpretations of the strength of the correlation are: small (0.1 ≤ |r| ≤ 0.29), moderate (0.3 ≤ |r| ≤ 0.49), large (0.5 ≤ |r| ≤ 0.69), very large (0.7 ≤ |r| ≤ 0.89), nearly perfect (0.9 ≤ |r| ≤ 0.99), perfect (|r| = 1.0) [32]. This interpretation is the interpretation of the strength of the correlation between 3MBT and Yo-Yo IRT.

Procedure

As a first action, all participants who voluntarily agreed to participate in the study (non-probabilistic sample) were invited and gathered for an informed talk. During the informed talk, the purpose and procedures of the study were clarified. Inclusion criteria for all participants were healthy and physically active, aged 18-25 years, and exclusion criteria were a positive COVID-19 diagnosis, the prevalence of musculoskeletal injury, abnormal respiratory and cardiac responses during the acclimation period, and an inability to perform 3MBT. All participants were asked to refrain from physical activity that produced neurological or musculoskeletal fatigue for 24 hours prior to the measurement. Finally, only participants who agreed to give informed consent were eligible for 3MBT.

This study was conducted at Momoyama Gakuin University from April 2022 to August 2022. This study was approved by the Research Ethics Committee of Momoyama Gakuin University (Reference No. 2020-07). Young male subjects with exercise habits were included in the study, and informed consent was obtained from all of them.

## Results

A total of 127 young men (mean age 18.96 ± 0.5 years, mean weight 61.53 ± 7.6 kg, mean body fat 20.44 ± 6.2 %, mean muscle mass 46.07 ± 5.9 kg) with a history of exercise for at least 90 minutes once a week were studied. The physical characteristics of the subjects were as follows (Table 1). The body mass index (BMI) was within the norm for male participants (mean BMI 21.96 ± 3.1 kg/m^2^).

**Table 1:** Body composition characteristics of study subjects BMR: basal metabolic rate

	WEIGHT （kg）	BODY FAT （％）	BMR （kcal）	BONE MASS （kg）	BODY WATER （％）	BMI （kg / m2）
Mean±SD	61.53±7.6	20.44±6.2	1460.45±143.4	2.60±0.3	53.53±4.2	21.96±3.1
	(39.7÷101.7）	(7.8÷40.0）	(1107.0÷2267.0）	(1.9÷3.9）	(42.0÷64.3）	(17.0÷32.7）

In addition, the skeletal muscle mass by region is given in Table 2.

**Table 2:** Skeletal muscle mass by subject region MUSCLE - M (muscle mass), MM L - ARM (left arm muscle mass), MM R - ARM (right arm muscle mass), MM L - LEG (left leg muscle mass), MM R - LEG (right leg muscle mass), MM TRUNK (trunk muscle mass)

	MUSCLE - M (kg)	MM L - ARM (kg)	MM R - ARM (kg)	MM L - LEG (kg)	MM R - LEG (kg)	MM TRUNK (kg)
Mean±SD	46.07±5.9	2.18±0.3	2.28±0.4	8.84±1.1	9.00±1.0	24.19±3.1
	(33.0÷72.4)	(1.3÷3.3)	(1.4÷3.5)	(3.0÷14.7)	(6.1÷14.7)	(17.2÷36.3)

**Figure 4:**
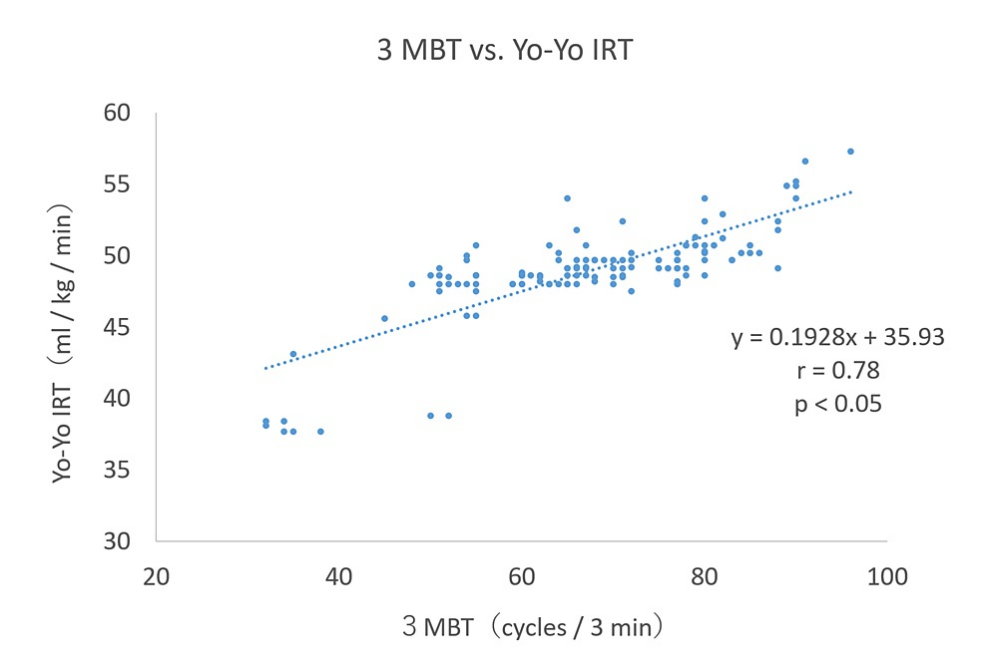
Relationship between 3MBT and Yo-Yo IRT 3MBT: 3-minute burpee test; Yo-Yo IRT: Yo-Yo intermittent recovery test

The estimated equation maximal oxygen uptake at Yo-Yo IRT averaged at 48.82 ± 2.0 ml/kg/min, with a maximum of 57.3 ml/kg/min. The 3MBT averaged 66.8 ± 10.8 cycles/3 min, with a maximum recorded at 96 cycles/3 min. There was a very large positive relationship between the estimated equation maximal oxygen uptake and 3MBT in Yo-Yo IRT (Figure 4).

## Discussion

Various tests using burpees have been developed, including 10, 20, 30, and 60-second tests [24,29,33]. While it was clear that there was a relationship between the number of burpee cycles performed, primarily with instantaneous force at short durations of 10 to 20 seconds and with a lean body mass at 30 to 60 seconds, the relationship with maximal oxygen uptake was not yet clear.

Maximal oxygen uptake represents the physiological upper limit of oxygen utilization to generate energy during intense exercise performed to voluntary exhaustion. Although exercise loading methods using large muscle groups of the whole body can produce very similar results to exercise loading methods using mainly lower limb muscle groups, the reason why the relationship with maximal oxygen uptake has not been clarified is that the burpee test has been performed for too short a time so far, and this was investigated.

The results of the study showed a very large positive relationship (r = 0.78) between the estimated formula for maximal oxygen uptake using the Yo-Yo IRT and the number of cycles of 3MBT, indicating that exercise loading using whole-body muscle groups is very similar to exercise loading methods using mainly the lower extremity muscle groups.

Although Yo-Yo IRT is normally used as a performance-enhancing measure rather than an estimation of maximal oxygen uptake, it was employed in this study as one that could be investigated during the coronavirus disease 2019 (COVID-19) pandemic without the need for special equipment. In the future, a more accurate method should be used to investigate the relationship with maximal oxygen uptake.

The burpee exercise in this study is also considered to be a whole-body exercise that uses not only the lower extremity muscle groups but also the large muscle groups of the upper extremities, such as the pectoralis major, rectus abdominis, and deltoid muscles, since squats and planks are performed from a standing posture, and thus the relationship is considered to be quite similar to the field test using mainly the lower extremity muscles.

Burpees are a series of push-up (push-up) exercises, but after a 3MBT in a preliminary experiment, push-ups were excluded because some could not be performed due to differences in push-up technique (upper limb lowering) and the number of times they were performed.

The results of the study showed a very large positive relationship (r = 0.78) between the estimated formula for maximal oxygen uptake using the Yo-Yo IRT and the number of cycles of the 3MBT, indicating that exercise loading using whole-body muscle groups is very similar to exercise loading methods using mainly the lower extremity muscle groups.

The burpee exercise in this study is also considered to be a whole-body exercise that uses not only the lower extremity muscle groups but also the large muscle groups of the upper extremities, such as the pectoralis major, rectus abdominis, and deltoid muscles, since squats and planks are performed from a standing posture, and thus the relationship is considered to be quite similar to the field test using mainly the lower extremity muscles.

Although the burpee is essentially a push-up and jump-up exercise in a series of movements, it was excluded for three reasons: first, as a result of a 3MBT in a preliminary experiment, a number of push-ups could not be performed due to differences in the push-up technique (upper limb lowering) or after a number of times The second reason was that the jump height of the jump-up was not specified and there were large individual differences, and the third reason was to make the method consistent with the methods of previous studies [34].

Further investigation is needed in a variety of populations, as more than half the subjects in this study had tennis experience, and the fact that the Yo-Yo IRT's intermittent exercise and repeated short rests are similar to the competitive characteristics of tennis is likely one of the factors that led to the strong relationship observed.

Recently, a study of the relationship between muscular endurance and risk of disease and life expectancy found that firefighters who could do 41 or more push-ups at baseline had a 96% lower risk of heart disease than those who could do fewer than 10, making it a more accurate predictor of heart disease than treadmill testing.

In addition, a study of the health status of 140,000 people in 17 countries found that for every 5 kg decrease in grip strength, there was a 17% increase in all-cause mortality rates, including cancer and heart disease, and statistical analysis of health data for the elderly from 50 previous studies found that the stronger the grip, the lower the mortality rate, the lower the probability of becoming bedridden, the lower the cognitive decline, the faster the recovery from illness. and the faster the recovery from illness [35-37].

These factors have led to recent reports that investigations using muscle endurance are beneficial to health.

In this study, the 3MBT was found to be related to maximal oxygen uptake as estimated using Yo-Yo IRT. The 3MBT is a field test that can be performed anytime and anywhere there is space for a plank or standing posture.

The limitations of this study, however, are that only young men with a history of exercise were included and that maximal oxygen uptake is an estimation equation.

Future field tests of maximal oxygen uptake in subjects of various ages, genders, and exercise histories should be conducted to further investigate the relationship. The 3MBT is fast and economical because it does not require any equipment. It can be performed in a small space, but as with exercise stress testing, the intensity is very high, and care must be taken to ensure that people at high risk of disease and the elderly are examined by a physician before performing the test.

## Conclusions

The present study showed that there is a strong relationship between 3MBT and the estimation of maximal oxygen uptake using the Yo-Yo IRT equation. This is because, in the past, the estimation of maximal oxygen uptake required a large space and equipment. However, as a result of this study, it can be easily performed if there is space for a clock and crunch posture. In addition, an improvement in the number of cycles is likely to lead to an improvement in maximal oxygen uptake, as it is related to maximal oxygen uptake. This information would be useful for a wide variety of people, not only athletes and healthy individuals. The information would also be useful in many educational institutions and sports settings because it can be assumed by many people simultaneously in a short period of time. The 3MBT would be useful not only as a new field test that does not require equipment or space but also as a training tool for athletes and able-bodied individuals to use muscle groups throughout the body.
